# Under pressure: personality, behavioural and physiological responses to handling in captive *Addax nasomaculatus* antelopes

**DOI:** 10.1098/rsos.260738

**Published:** 2026-10-28

**Authors:** Matías Villagrán Boerr, César Echaides, Martin W. Seltmann, Vedrana Šlipogor, Bruno Esattore, Laura Saggiomo, Juan Pablo Damián

**Affiliations:** ^1^ Departamento de Biociencias Veterinarias, Facultad de Veterinaria, Universidad de la República, Montevideo, Uruguay; ^2^ Núcleo de Desarrollo Académico en Medicina de la Conservación, Universidad de la República, Facultad de Veterinaria, Montevideo, Uruguay; ^3^ Parque Villa Dolores, Intendencia de Montevideo, Montevideo, Uruguay; ^4^ Department of Biology, University of Turku, Turku, Finland; ^5^ Department of Ecology and Evolution, University of Lausanne, Lausanne, Switzerland; ^6^ Institute of Microbiology, University of Lausanne, Lausanne, Switzerland; ^7^ The Sense Research and Innovation Centre, Lausanne, Switzerland; ^8^ Department of Wildlife, Fish, and Environmental Studies, Swedish University of Agricultural Sciences, Uppsala, Sweden; ^9^ Núcleo de Bienestar Animal, Universidad de la República, Facultad de Veterinaria, Montevideo, Uruguay

**Keywords:** coping style, temperament, husbandry, ungulate, endangered

## Abstract

Animal personality refers to consistent behavioural differences among individuals across time and contexts. Knowledge of personality in ruminants, particularly non-domesticated species, remains limited. The study aimed to assess personality in a herd of *Addax nasomaculatus* antelopes (*n* = 25) living in a zoo, via a questionnaire, and to examine its relationship with handling responses under captive conditions. Personality was assessed using a 20-item questionnaire rated by observers across three contexts (Feeding, Neutral and Handling). Handling responses were measured via surface temperature, feeding and locomotor behaviour (pre- and post-handling), exit velocity and faecal cortisol metabolites. Data were analysed using exploratory factor analysis and linear mixed models. Four personality dimensions were identified: ‘Active/Unpredictable’, ‘Aggressive/Excitable’, ‘Bully’ and ‘Confident’. These traits were associated with distinct handling responses, indicating consistent interindividual variation in stress responses. Specifically, individuals scoring higher in ‘Aggressive/Excitable’ showed higher exit velocity and lower surface temperature, whereas those scoring higher in ‘Active/Unpredictable’ and ‘Bully’ showed lower exit velocity, higher surface temperature and increased grazing after handling. No association was found between personality and faecal cortisol metabolites. These findings expand the limited knowledge on non-domestic ruminants and have implications for improving captive management and conservation programmes.

## 1. Introduction

Animal personality is defined as consistent behavioural differences among individuals, maintained across time and contexts [1,2]. Such behavioural consistency is expected within individuals, both across repeated exposures to the same stimulus and across responses to different stimuli [3]. Personality shapes behavioural tendencies, including foraging [4–6], and encompasses personality traits such as Aggressiveness [7]. It is also relevant for many aspects of species biology [8], including animal health [9,10] and welfare [11]. In addition, personality can affect the success of reintroducing wildlife from captivity to the wild [12–14].

Currently, the axes most frequently used to quantify personality across taxa include shyness–boldness, exploration–avoidance, activity, aggressiveness and sociability [2,15,16]. In addition to these, individuals have also been classified as proactive or reactive in the literature, based on their interindividual differences in the behavioural and physiological strategies used to cope with challenging or stressful situations (i.e. coping style) [17]. Stress responses involve an early activation of the sympathetic–adrenomedullary (SAM) axis, promoting catecholamine release and a fight-or-flight response, typically accompanied by parasympathetic withdrawal [18–20]. Subsequent activation of the hypothalamic–pituitary–adrenal (HPA) axis leads to cortisol secretion from the cortex of the adrenal gland [19,21]. Proactive individuals typically exhibit higher aggression, routine formation and active coping strategies when confronted with challenges, often displaying a fight-or-flight type behavioural response [20,22,23]. In contrast, reactive individuals are characterized by greater behavioural flexibility, lower aggression and a tendency to respond to challenges through more passive or cautious coping strategies [19,24]. Importantly, coping style and stress reactivity are now considered related but partially independent dimensions of individual variation, with the former reflecting consistent behavioural strategies and the latter describing the magnitude and dynamics of physiological stress response [22,23]. For individuals living in the wild, their stress response is often adaptive, as it helps the animal to quickly assess their situation and to prepare adequate behavioural reactions, while also providing the necessary energy for a sudden increase in motor activity [19,24,25]. In captivity, however, stress reactions may be caused by limited habitats with less options to express their full movement, living in groups of abnormal social structure and being more often exposed to artificial lights and sounds, and constant human proximity [26,27].

Management of non-domestic animals under human care aims to safeguard their health and welfare [28]. Nevertheless, captive housing can lead to negative physiological outcomes [27], as stressors, such as confinement, human presence and an unfamiliar environment, may affect animals and, consequently, their responses [26]. Thus, routine husbandry procedures (e.g. handling) may elicit stress and impaired welfare [29,30], potentially contributing to immunosuppression, disease and reduced reproductive performance [19,21,31–34].

With approximately 200 extant species, ruminants are an important zoological group for agriculture, conservation and biomedicine [35,36]. They also represent a substantial proportion of the global biomass of both domestic and non-domestic terrestrial mammals, underscoring their ecological and economic importance [37]. Despite this, knowledge on personality in ruminants, and especially non-domesticated species, remains limited. Deer are the most studied group among ungulates (approx. 30 studies on seven species, reviewed in [3]). Some studies assessing non-domestic ungulate coping styles report higher cortisol concentrations in reactive individuals, consistent with greater HPA axis activation (guanaco, *Lama guanicoe* [38]; roe deer, *Capreolus capreolus* [39]), whereas others suggest the opposite pattern (fallow deer, *Dama dama* [40]). Evidence in antelopes is particularly scarce and is restricted to a single study in common eland (*Taurotragus oryx*), in which more reactive individuals during handling showed higher albumin, globulins, total protein, glucose, alkaline phosphatase and lactate dehydrogenase levels in plasma, suggesting a stronger stress response [41]. Aiming to fill the gap on the link between personality and handling responses in non-domestic ruminants, this study focuses on the addax antelope (*Addax nasomaculatus*), a native inhabitant of the desert and semi-desert regions of the African continent [42–44]. The species is currently critically endangered and considered critically depleted according to the recovery conservation perspectives [43,45]. Latest reports estimate fewer than 100 adult individuals in the wild, limited to a few patches of desert in Niger and Chad, and approximately 5000 individuals living in captivity [43,46]. These captive populations are distributed across conservation breeding programmes, zoological institutions and managed reserves worldwide [45] and have played a central role in preventing the species’ complete extinction, through, for example, the reintroduction of specimens into the wild [47]. The dramatic decline of wild populations has been mainly attributed to overhunting, habitat degradation, competition with livestock and political instability across its historical range. In response, several conservation actions have been implemented, including captive breeding programmes, international management of *ex situ* populations and reintroduction initiatives aimed at restoring free-ranging populations in protected areas. Despite the serious risk of extinction faced by the species, scientific knowledge about it is extremely limited. Specifically, the potential consequences of common handling procedures in captivity on their stress, as well as their interindividual differences in responses to these conditions, remain largely unexplored in this species. Studying the general pattern of management-related stress response and its interindividual differences seems critical to improve management of captive specimens, help optimize pre-reintroduction training and improve post-release survival and adaptation in this critically endangered ungulate species [13,14]. In addition, characterizing these aspects allows us to consider personality as a source of individual variation that should be maintained and promoted both in *ex situ* breeding programmes and *in situ* conservation efforts.

In line with previous findings in non-domestic ruminants, we predicted describing distinct personality traits associated to aggressiveness, activity, boldness–confidence and sociability in the addax. We further predicted interindividual differences in the response to handling, according to personality traits. Specifically, we predicted that individuals with higher aggressiveness scores show greater reactivity to manipulation than those with lower scores, whereas the opposite pattern would occur for active, social and confident personality traits. Therefore, with the overall goal of improving our understanding of handling-related responses in addax, this study aimed to assess personality in a herd of adult addax antelopes living in a zoo via a questionnaire and to establish how their personality relates to behavioural and physiological handling responses under captive conditions.

## 2. Material and methods

### 2.1. Experimental site and animals

The study was conducted with the addax antelope population housed at the Parque Lecocq Zoo (Montevideo, Uruguay; 34°47′ S, 56°20′ W). Our study subjects included 25 animals, 15 adult females (8.8 ± 1.5 years) and 10 adult males (9.4 ± 1.2 years), allocated in single-sex groups that remained stable in composition throughout the study. Males were distributed in three paddocks (approx. 0.25 ha each) and females in one paddock (0.5 ha). All paddocks were covered by native pasture, included trees, feeders and a water reservoir, and were connected to a 25 m^2^ roofed shelter.

Animals were fed native pasture, alfalfa (*Medicago sativa* L.) hay and commercial ration formulated for antelopes (Nutrisur, Canelones, Uruguay; protein: 17%, fibre: 5%, maximum humidity: 13%), which was offered daily. Animals were individually recognizable by ear tags, microchips and morphological traits, including the size and shape of their horns. All animals were habituated to human presence and to the use of the handling facilities (see description below), as part of the zoo's veterinary management.

### 2.2. Animal handling

The individuals were handled to record their individual response to this procedure, which occurred once per season over a 1-year period. For each seasonal sampling, handling was conducted within a 2-day window, between 8:00 and 14:00 h. As a result, each animal was handled four times during the study. Subgroups of a maximum of 10 animals from the same social group were successively moved to the covered shelter, remaining in a waiting room until individually handled in a crush (i.e. a mechanical restraining stall). Total time in the crush on average was 11.9 ± 0.3 min, and the procedure was consistent across individuals and handlings. The procedure started 1 min after the animal had entered the crush.

The animal was held by its horns by two handlers, while hair samples from the shoulder and rump (10 × 10 cm each) were taken using an electric hair clipper, as part of an independent study. Subsequently, the animal's surface temperature was determined using a thermal camera (FLIR E95, FLIR Systems, Estonia), with a temperature range of −20 to +120°C and an accuracy of ±0.3°C. The camera was set to an emissivity of 0.98, as reported in previous studies for measurements of skin and ocular surface temperature [48–50], and had a field of view of 80° × 60° using a 2 mm lens. The individual remained in the crush for another minute without handling to calm down and was subsequently released, remaining physically isolated from conspecifics for 5 min in a separate paddock. Finally, the individual returned to its group. The time spent in the crush and the 5 minutes after releasing were recorded on independent videos and were used to assess personality. The exit from the crush was videorecorded, and the exit velocity [51] was calculated as the time required to cover 3 m distance after release.

### 2.3. Personality assessment

The trait rating technique [52–54] was applied to a specifically constructed questionnaire for the addax antelope by using a combination of the bottom-up as well as the top-down approaches. Briefly, top-down approaches to animal personality assessment define personality traits *a priori* based on theoretical frameworks derived largely from human psychology and evaluate them through standardized trait descriptors or rating questionnaires applied by experienced observers [1,52,55]. Meanwhile, the bottom-up approaches derive personality dimensions inductively from repeated observations of consistent behavioural patterns, allowing traits to emerge directly from species-specific behavioural repertoires and ecologically relevant contexts without assuming predefined personality structures [2,53,56]. Based on several personality questionnaires from different mammals and considering the behavioural locomotor, feeding and social repertoire of the addax [57–59], the following items for the species were selected: Erratic (primates [60]), Active (deer [4,54]), Calm/Docile (spotted hyena, *Crocuta crocuta* [1]) and Friendly/Social (Asian elephant, *Elephas maximus* [61]). As a result, the questionnaire included 20 items (table 1). These constituted the initial set of questionnaire items. As described below, observer ratings of these items were subsequently analysed to identify underlying personality dimensions based on the relationships among the descriptors.

**Table 1.** Description and inter-rater consistency (intra-class correlation coefficient; ICC) of items initially considered for the construction of personality axes in the captive adult addax (*Addax nasomaculatus*) antelope.

trait	description	ICC(3,1)	ICC(3,k)
Active	the antelope moves around a lot, often at a fast pace, and spends little time being still	0.139	0.862
Socially Anxious	the antelope seems to be restless when it is close to another antelope; it carefully observes the social surroundings	0.024	0.487
Aggressive	the antelope often initiates physical fights or conflicts with others, which causes harm or potential harm to other individuals, both animal and human	0.067	0.735
Bully	an antelope is overbearing and initiating a confrontation towards younger, smaller or of lower rank animal	0.041	0.620
Calm/Docile	the antelope does not get easily excited and reacts to change in a relaxed, unhurried way	0.167	0.885
Confident	the antelope behaves in an assured manner; it makes quick decisions and does not hesitate; it initiates the interactions and displaces other antelope	0.044	0.641
Distractible	the antelope easily loses focus, has a short attention span and does not focus long on activities	0.022	0.461
Erratic	the individual is inconsistent, indefinite and widely varying in its behaviour and moods	0.047	0.653
Excitable	the antelope overreacts to any change, gets easily excited from outside disturbances and does not calm down easily	0.092	0.795
Inquisitive	the antelope readily explores new situations, objects, animals or people and tries to learn new things	0.064	0.724
Popular	the antelope is sought out as a companion by others, those who actively seek to contact with it	0.059	0.706
Predictable	the antelope's behaviour is consistent and steady over extended periods of time; it does little that is unexpected or deviates from its usual behavioural routine	0.017	0.398
Slow/Nonactive	the antelope moves and rests in a relaxed manner, moving slowly and deliberately, not easily hurried	0.143	0.865
Submissive	the antelope gives in readily to others of a similar size and acts as though lower in rank to other antelopes; for example, they will retreat or turn away in interactions	0.150	0.872
Persistent	the antelope does not give up easily on some activity	0.082	0.774
Vigilant	the antelope carefully watches for possible dangers in the surroundings	0.089	0.790
Friendly/Social^a^	the antelope initiates close contact to others within their group (scratching, rubbing, etc.) or lies/stands close to others (<2 m) while resting	0.085	0.782
Solitary^a^	the antelope prefers to spend time alone and does not seek out contact with other individuals	0.079	0.769
Protective^a^	antelope often intervenes to prevent harm or annoyance from coming to others	0.034	0.576
Fearful^b^	the antelope reacts to threatening objects/situations in a frightened manner	0.033	0.567

The list of items was constructed taking into account the behavioural pattern described for the species [57–59] and items used in other species (e.g. [62–64]). Single-measure reliability is reflected by ICC(3,1), and the reliability of the average of k ratings is reflected by ICC(3,k).

Items not included in the analysis (exploratory factor analysis) owing to low values in the measure of sampling adequacy (<0.7; ^a^) or possible Heywood cases (i.e. improper parameter estimates in the latent variable model) (^b^).

Personality was assessed during the 1-year period that the study lasted and included three different conditions: Feeding, Neutral and Handling. The Feeding condition included the 2 h immediately after offering supplement. This interval was selected because the animals showed the greatest interest in and presence at the feeders during this period and were generally not observed resting or engaging in activities unrelated to feeding in the vicinity. The Neutral condition was a period without voluntary anthropic stimuli and feeding (at least 3 h after receiving the concentrate and after removing any amount of it from the feeders). For the Feeding and Neutral conditions, three 15 min videos per individual were recorded, each on different days (i.e. repetitions). For the Feeding condition, the start time of each 15 min video was randomized within the 2 h window following food delivery. Two observers using separate cameras recorded videos simultaneously. When multiple individuals were captured in the same video, each individual was subsequently assessed independently. The Handling condition included the manipulations performed on each per season. Videos about it included the time spent in the crush and the time in the releasing paddock (see description above).

Videos of each individual in each condition (three videos for Feeding and Neutral and four videos for Handling) were independently analysed to rate the questionnaire traits. Four raters with a minimum of 18 months of previous experience in observing and studying the behaviour of addax antelopes and these particular individuals filled in the questionnaire independently for all individuals across all conditions. A 1–5 scale was used to describe the propensity of each animal to display a specific trait (1 = ‘almost never’, 2 = ‘very rarely’, 3 = ‘occasionally’, 4 = ‘quite a lot’, 5 = ‘most of the time’). Raters were previously instructed not to exchange information about their assessments with each other.

### 2.4. Response to handling

#### 2.4.1. Surface temperature

While the animal remained in the crush, one thermal image of the eye, shoulder and rump was taken to estimate both autonomic responses and changes in whole-body heat load and dissipation [62,65,66]. Three measurements were done from each picture, and the average surface temperatures for each body part were subsequently calculated.

#### 2.4.2. Behaviour

We used the scan sampling method [63] by direct visual inspection, every 10 min, for 8 h per day (8.00–12.00 and 13.00–17.00 h), two days before and two days after each handling (‘pre-handling’ and ‘post-handling’, respectively) to collect behavioural data [58]. The feeding behaviour (grazing, eating concentrate and ruminating) and locomotor behaviour (standing, lying and walking) performed by each individual were recorded on paper forms. The frequency of all these behaviours was calculated as the percentage of the total daily observations in which the animal performed each behaviour. The magnitude of change of each behaviour associated with handling was calculated as post-handling behaviour: pre-handling behaviour/pre-handling behaviour × 100. Thus, when the usage time of a behaviour after manipulation was less than before it, the percentage change value resulted in a negative value.

#### 2.4.3. Faecal cortisol metabolites

Faeces from known individuals were collected immediately after defecation, within 48 h before manipulation, and within 48 h after manipulation, and frozen at −20°C until processing. The extraction of faecal cortisol metabolite (FCM) was performed according to Palme *et al.* [67]. Briefly, approximately 3 g of each sample was dehydrated at 60°C for 48 h and subsequently pulverized using a mortar. From it, 0.5 ± 0.003 g of faeces were weighed, 5 ml of 80% methanol was added, and the mixture was shaken for 12 h and centrifuged. The supernatant was extracted and placed in a clean sealed tube at −20°C until hormonal determination. A 100 µl fraction of this solution was used to determine the FCM concentration. A subset of samples was randomly selected, and serial dilutions (between 1:2 and 1:32 using 1% PBS) were done to establish the most suitable dilution for hormonal determination. The FCM concentration was determined by an ELISA kit (Cortisol Elisa Equipment; Neogen Corporation, Ayr, UK). The intra-assay coefficient of variation was 7.3 ± 3.3, the inter-assay coefficient was 9.3 ± 2.3, and the linearity of the dilutions showed a correlation of *R*^2^ = 0.99. The detection range was 0.04–10.0 ng ml^−1^. The magnitude of change in FCM concentration according to handling was calculated as follows: post-handling concentration/pre-handling concentration × 100.

### 2.5. Statistical analyses

Data were analysed with the Rv.4.3.2 software (R Core Team, 2023).

#### 2.5.1. Personality assessment

One thousand questionnaires were assessed throughout the study, resulting from four raters evaluating 25 addax individuals under three repeated conditions: three times for Neutral and Feeding conditions and four times for Handling condition. The ‘Bully’, ‘Popular’, ‘Protective’ and ‘Solitary’ traits were not rated during the Handling condition as these items required interaction with another individual, which was not possible during individual manipulations. In those cases, the missing values (*n* = 396) were replaced with the midpoint of the personality scale, that is ‘3’. The total number of ratings was 19,604. For each individual, the mean score of each item was calculated considering conditions and repetitions.

To determine the reliability of items between raters, the intra-class correlation coefficient (ICC) was estimated for each item, across all three conditions together, using both ICC(3,1), which reflects single-measure reliability, and ICC(3,k), which reflects the reliability of the average of k ratings [68]. As a preliminary screening step, items showing positive ICC values were retained for subsequent evaluation through the factorial analyses. The average of the ratings per item given by the four observers was calculated. To test the sampling adequacy of the correlation matrix, we performed the Bartlett sphericity test and the Kaiser–Meyer–Olkin (KMO) measure of sampling adequacy (all functions from the package *psych*, v.5.0.0 [69]. The Bartlett sphericity test was significant (*p* < 0.01), showing that the correlation matrix was significantly different from a matrix in which traits are not correlated [70]. However, the examination of the KMO test showed that the overall measure of sampling adequacy (MSA) value was 0.64 and therefore somewhat lower than the recommended value of 0.7 [70]. In addition, several items had rather low MSA values, and therefore, it was decided to remove those from further analyses (‘Protective’, MSA = 0.36; ‘Friendly/Social’, MSA = 0.45; ‘Solitary’, MSA = 0.46). Furthermore, preliminary analysis had shown that the item ‘Fearful’ caused potential Heywood cases (i.e. improper parameter estimates in the latent variable model), and it was therefore removed from further analyses as well. Then, the KMO test was run again, and the overall MSA value reached the threshold of 0.7. Three approaches to assess the number of factors to retain in the factor solution [71] were combined: (i) eigenvalues >1, (ii) scree plot analysis, and (iii) Horn's parallel analysis with 10 000 iterations (package *paran* [72]). Then, an EFA (exploratory factor analysis; function *fa* from package *psych* [69]) was performed, using a maximum likelihood approach and 1000 iterations to investigate how the remaining items were associated with each other as factors. Finally, the factor scores for each individual addax antelope were extracted for the four personality axes (see description in ‘Results’). In the EFA, communality (H²) was used to assess the proportion of variance in each trait accounted for by the extracted factors.

#### 2.5.2. Response to handling

When analysing the response variables, we first evaluated data distribution (function *fitdist*, package *fitdistrplus*). The variables ‘change in FCM concentration’ and ‘exit velocity’ were log-transformed (natural logarithm transformation) to improve the normality of the residuals and to reduce skewness. Linear mixed models were constructed (function *lmer*, package *lme4*), to relate personality axes with the percentage magnitude of change in feeding behaviour (grazing, eating concentrate and ruminating), locomotor behaviour (standing, lying and walking) and FCM, together with the exit velocity and surface temperatures (eye, shoulder and rump) in relation to handling. Independent models were defined to examine the relationship between personality axes and each response variable. A repeated measures structure was used, with personality axes as fixed effects and individuals as random effects. Given the seasonal variation in behaviour [58] and hair cortisol [73] within the same addax antelope population, the season in which handlings were performed was included as a covariate. In light of the study objectives, the results concerning this effect are briefly reported in ‘Results’ but will not be discussed in detail. The age and sex of the animals were initially included in the models as potential effects. However, since these factors did not improve the model fit, they were removed from the final models. Following the approach of Burnham and Anderson [74], the selection of the final models was based on Akaike's information criterion (AIC) [75]. The goodness of fit of each model was checked by visual inspection of the residuals, including the presence of influential observations and overdispersion. Significant effects were considered when *α* ≤ 0.05 and a tendency when 0.1 ≥ *α* > 0.05. Predicted effects of personality factors on the exit velocity, surface body temperatures and the magnitude of change in grazing behaviour were obtained using the *ggpredict* function (package *ggeffects*). Data are presented as mean ± s.e.m.

## 3. Results

### 3.1. Personality assessment

All items showed positive ICC values, ranging from 0.017 to 0.167 for the ICC (3,1) and ranging from 0.398 to 0.885 for ICC (3,k) (table 1). The combined results from the scree plot test, with eigenvalues >1, and a Horn's parallel analysis test with 10 000 iterations suggested factors for the remaining 16 items. An EFA solution was oblimin rotation was performed, and loadings >±0.4 were considered salient. Considering the value and direction (positive/negative) of each item, the factors were labelled ‘Active/Unpredictable’, ‘Aggressive/Excitable’, ‘Bully’ and ‘Confident’. For example, the Active trait loaded the highest and positive value, thus defining the name of factor ‘Active/Unpredictable’ (table 2).

**Table 2.** Correlation matrix between behavioural traits and personality factor axes in captive adult addax (*Addax nasomaculatus*) antelopes.

trait	‘Active/Unpredictable’	‘Aggressive/Excitable’	‘Bully’	‘Confident’	H^2^
Active	**0.94**	0.10	0.11	0.01	1.00
Inquisitive	**0.80**	−0.09	−0.15	0.29	0.81
Slow/Nonactive	−**0.78**	−0.27	−0.09	0.05	0.95
Predictable	−**0.62**	−0.21	0.20	−0.25	0.80
Erratic	**0.61**	0.28	−0.30	0.00	0.80
Aggressive	−0.10	**0.86**	0.31	−0.05	0.90
Calm/Docile	−0.31	−**0.77**	0.01	0.07	1.00
Submissive	−0.06	−**0.70**	0.12	−**0.50**	0.78
Excitable	0.29	**0.66**	0.03	−0.27	0.82
Persistent	0.35	**0.57**	−0.23	0.12	0.76
Vigilant	0.38	**0.42**	0.18	−0.19	0.60
Bully	0.17	0.09	**0.87**	0.19	0.78
Distractible	0.24	0.01	−**0.81**	0.10	0.84
Popular	−0.50	0.12	**0.56**	0.22	0.61
Confident	0.15	0.00	0.14	**0.98**	1.00
Socially Anxious	0.27	0.26	0.51	−**0.54**	0.85

*Note:* Communality (H²) indicates the proportion of variance in each trait that is explained by the extracted factors. Trait values that contributed most strongly to each personality factor axis are signed in bold. Exploratory factor analysis, using a maximum likelihood approach and 1000 iterations.

### 3.2. Response to handling

The effects of personality axes and season on the behavioural and physiological response variables are presented in table 3, including the regression coefficients (*β*), standard errors (s.e.m.), *f* statistics and *p*-values for each variable.

**Table 3.** Effects of personality axes and season on the response variable estimated using linear mixed models.

	‘Active/Unpredictable’	‘Aggressive/Excitable’	‘Bully’	‘Confident’	season
surface temperature					
eye	*β* = −0.1; s.e.m. = 0.3; *f* = 0.1; *p* = 0.70	*β* = −0.0; s.e.m. = 0.2; *f* = 0.1; *p* = 0.98	*β* = 0.5; s.e.m. = 0.2; *f* = 8.3; ***p* = 0.003**	*β* = −0.2; s.e.m. = 0.2; *f* = 0.1; *p* = 0.29	*f* = 39.8; ***p* ≤ 0.001**
shoulder	*β* = 1.9; s.e.m. = 1.1; *f* = 2.9; *p* = 0.08	*β* = −1.9; s.e.m. = 0.9; *f* = 2.7; ***p* = 0.05**	*β* = 2.4; s.e.m. = 0.8; *f* = 9.0; ***p* = 0.003**	*β* = −1.2; s.e.m. = 0.7; *f* = 0.6; *p* = 0.08	*f* = 115.5; ***p* ≤ 0.001**
rump	*β* = 1.3; s.e.m. = 1.3; *f* = 1.6; *p* = 0.32	*β* = −1.2; s.e.m. = 1.1; *f* = 0.6; *p* = 0.30	*β* = 1.9; s.e.m. = 0.9; *f* = 4.1; ***p* = 0.04**	*β* = −1.1; s.e.m. = 0.8; *f* = 0.4; *p* = 0.19	*f* = 106.7; ***p* ≤ 0.001**
exit velocity	*β* = −0.3; s.e.m. = 0.1; *f* = 0.2; ***p* = 0.004**	*β* = 0.4; s.e.m. = 0.1; *f* = 7.3; ***p* ≤ 0.001**	*β* = −0.2; s.e.m. = 0.1; *f* = 7.1; ***p* = 0.01**	*β* = −0.0; s.e.m. = 0.1; *f* = 0.9; *p* = 0.88	*f* = 1.5; *p* = 0.13
*magnitude of change (%)*					
grazing	*β* = 108.4; s.e.m. = 48.4; *f* = 2.9; ***p* = 0.02**	*β* = −66.4; s.e.m. = 42.3; *f* = 2.5; *p* = 0.11	*β* = −4.6; s.e.m. = 34.8; *f* = 0.0; *p* = 0.89	*β* = 1.8; s.e.m. = 30.2; *f* = 0.09; *p* = 0.95	*f* = 6.6; ***p* ≤ 0.001**
concentrate consumption	*β* = 16.1; s.e.m. = 14.8; *f* = 0.0; *p* = 0.28	*β* = −19.9; s.e.m. = 12.9; *f* = 1.9; *p* = 0.12	*β* = 16.0; s.e.m. = 10.7; *f* = 2.3; *p* = 0.13	*β* = −0.3; s.e.m. = 9.3; *f* = 0.2; *p* = 0.97	*f* = 1.9; *p* = 0.11
ruminating	*β* = −10.5; s.e.m. = 45.1; *f* = 0.0; *p* = 0.82	*β* = −0.9; s.e.m. = 39.5; *f* = 0.0; *p* = 0.98	*β* = 31.2; s.e.m. = 32.5; *f* = 0.9; *p* = 0.33	*β* = −34.4; s.e.m. = 28.2; *f* = 0.8; *p* = 0.22	*f* = 2.2; *p* = 0.09
lying	*β* = 4.9 s.e.m. = 11.2; *f* = 0.03; *p* = 0.66	*β* = −5.6; s.e.m. = 9.8; *f* = 0.4; *p* = 0.56	*β* = −1.8; s.e.m. = 8.1; *f* = 0.0; *p* = 0.82	*β* = −7.7; s.e.m. = 7.0; *f* = 1.8; *p* = 0.28	*f* = 1.5; *p* = 0.21
walking	*β* = −29.9; s.e.m. = 26.5; *f* = 1.9; *p* = 0.26	*β* = 11.9; s.e.m. = 23.2;*f* = 0.2; *p* = 0.61	*β* = −16.2; s.e.m. = 19.1; *f* = 0.7; *p* = 0.39	*β* = −8.2; s.e.m. = 16.6; *f* = 0.7; *p* = 0.62	*f* = 1.5; *p* = 0.18
standing	*β* = −5.1; s.e.m. = 5.2; *f* = 0.2; *p* = 0.32	*β* = 4.7; s.e.m. = 4.5; *f* = 1.0; *p* = 0.31	*β* = −0.6; s.e.m. = 3.7; *f* = 0.03; *p* = 0.86	*β* = 1.4; s.e.m. = 3.2; *f* = 0.3; *p* = 0.65	*f* = 0.7; *p* = 0.54
faecal cortisol metabolites	*β* = 0.1; s.e.m. = 0.1; *f* = 0.2; *p* = 0.49	*β* = −0.1; s.e.m. = 0.1; *f* = 0.0; *p* = 0.62	*β* = −0.0; s.e.m. = 0.1; *f* = 0.2; *p* = 0.99	*β* = 0.0; s.e.m. = 0.1; *f* = 0.0; *p* = 0.98	*f* = 5.5; ***p* ≤ 0.001**

*Note:* The table reports regression coefficients (*β*), standard errors (s.e.m.), *f* statistics and associated *p*-values for each fixed effect. Individual identity (ID) was included as a random factor in all models. Statistical differences are highlighted in bold.

Magnitude of change in grazing behaviour increased with the ‘Active/Unpredictable’ personality factor, and it varied across the seasons (figure 1). Specifically, the three individuals with the lowest ‘Active/Unpredictable’ personality scores (approx. 10% of the population) showed a negative percentage change in grazing behaviour, contrasted with a positive percentage change in the three highest-scoring individuals (−149.5 ± 72.1% vs. 71.9 ± 47.9%, respectively). The response in ruminating tended to vary with seasons but did not differ between personality axes. The variation in concentrate consumption, lying, walking and standing did not vary with personalities or between seasons.

**Figure 1.**
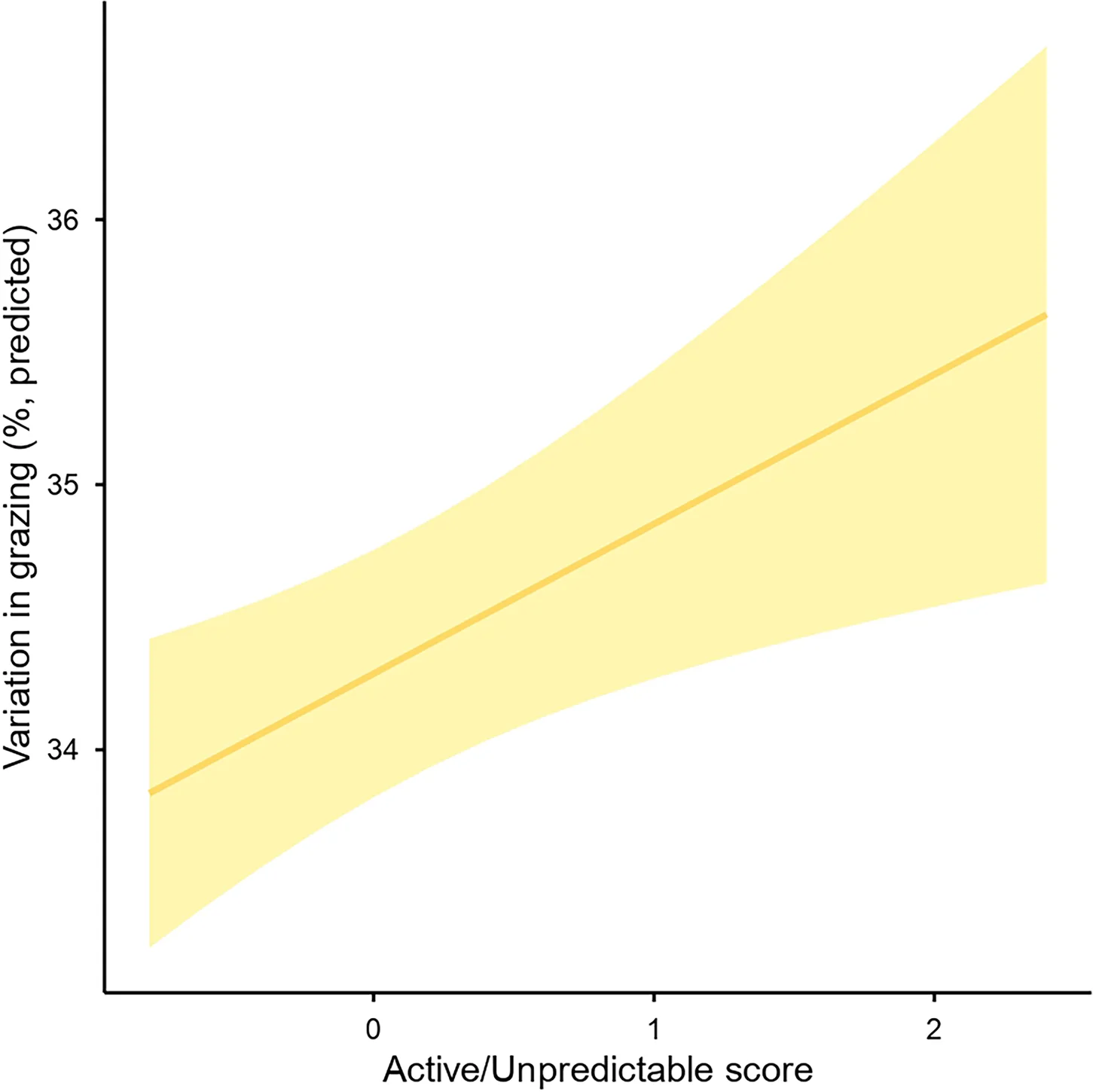
Magnitude (%) of change in grazing behaviour according to ‘Active/Unpredictable’ personality score in captive adult addax (*Addax nasomaculatus*) antelope.

The eye surface temperature increased with higher ‘Bully’ scores (figure 2a) but did not vary with the rest of the personality factors. The shoulder surface temperature increased with the ‘Bully’ scores (figure 2b) and decreased with increasing values of ‘Aggressive/Excitable’ scores (figure 2d). This temperature tended to increase with the ‘Active/Unpredictable’ and to decrease with the ‘Confident’ personality factors. The rump surface temperature increased as the ‘Bully’ factor score increased (figure 2c) but did not vary with the other personality factors. Surface temperatures at the eye, shoulder and rump varied with the seasons.

**Figure 2.**
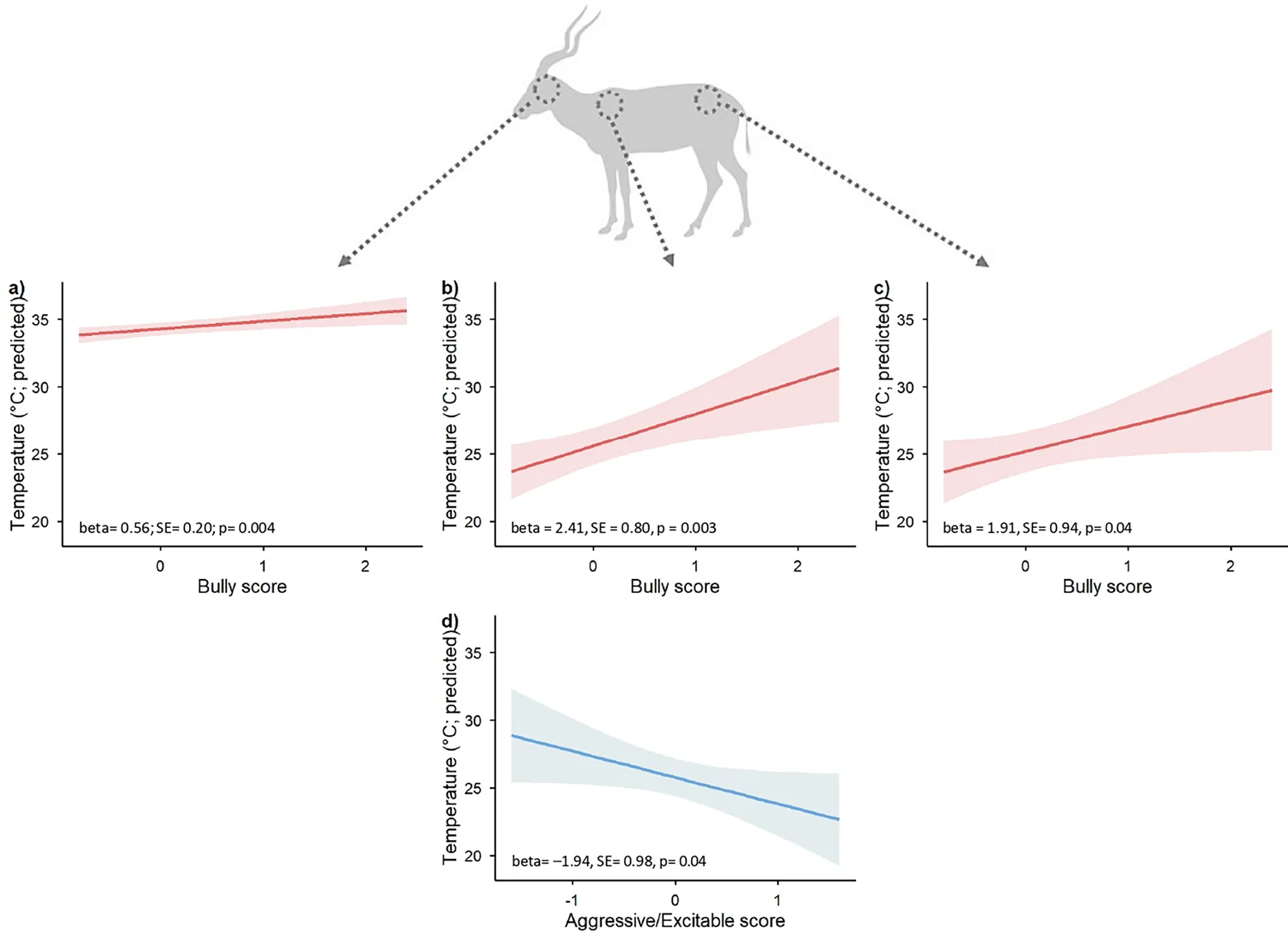
Body surface temperature at the (a) eye, (b,d) shoulder and (c) rump, according to (a–c) ‘Bully’ and (d) ‘Aggressive/Excitable’ personality axes in captive adult addax (*Addax nasomaculatus*) antelope.

Exit velocity increased with higher values of ‘Aggressive/Excitable’ and decreased with increasing values of ‘Active/Unpredictable’ and ‘Bully’ personality factor scores (figure 3a–c, respectively). There was no relationship between the ‘Confident’ personality factor and the exit velocity. This variable did not change with the season.

**Figure 3.**
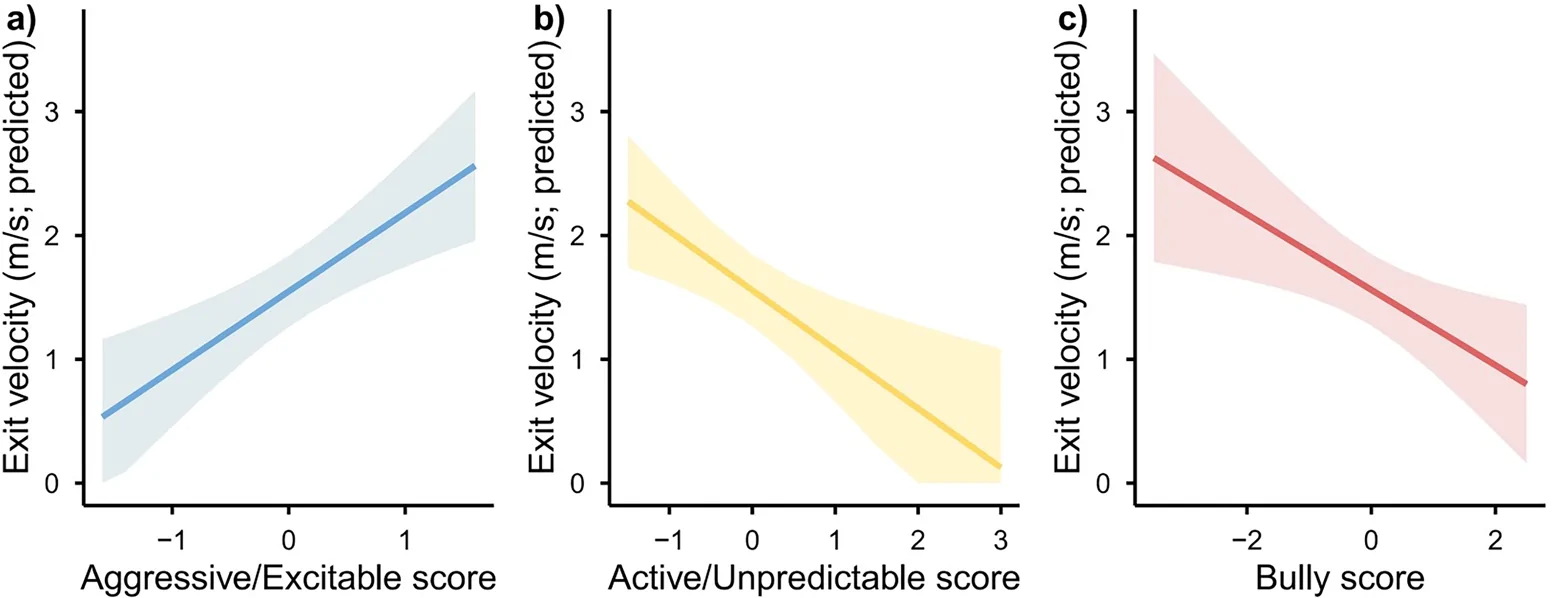
Exit velocity from the crush according to (a) ‘Aggressive/Excitable’, (b) ‘Active/Unpredictable’ and (c) ‘Bully’ personality axes in captive adult addax (*Addax nasomaculatus*) antelope. Shaded areas represent 95% confidence intervals, with the lower bound in panel (b) truncated to zero to prevent biologically unrealistic negative velocity values.

The variation in FCM in response to handling did not differ with personality scores, but it differed between seasons.

## 4. Discussion

This study provides the first description of personality axes in a captive population of adult addax antelopes and their relationship with behavioural and physiological responses to handling. In accordance with our first hypothesis, personality axes included Aggressiveness, Confidence, Activity and Sociability traits. Most personality axes were related to interindividual differences in behavioural and physiological response to handling. Higher ‘Aggressive/Excitable’ scores were linked to higher exit velocity and lower surface temperature, suggesting greater reactivity to handling, while higher scores of ‘Active/Unpredictable’ and ‘Bully’ axes were associated with lower exit velocity, higher grazing response and higher body surface temperature. These findings support our second hypothesis. Therefore, confirming the existence of four personality axes in the addax antelope and their link to stress responses fulfils our primary objective: improving the understanding of how this species responds to handling.

We used a carefully constructed questionnaire to assess personality in this species, and it included 20 behavioural items, with some that were not previously described for the species (e.g. ‘Inquisitive’ and ‘Predictable’). The 16 retained items showed varying levels of inter-rater consistency, but their contribution to a coherent four-factor structure supported their use in characterising personality in this population. The factorial structure further suggests that potential temporal instability of traits [76,77] or ambiguity or overlap among trait definitions [78] did not substantially compromise the identification of distinct personality dimensions. In contrast, ‘Protective’, ‘Friendly/Social’, ‘Solitary’ and ‘Fearful’ showed insufficient agreement and were excluded from the factorial analysis, likely owing to their context dependence and limitations in their expression under some of the study conditions. Fearfulness is known to vary in response to novel objects and human presence [76,79], both of which differed across our conditions (e.g. close human presence and exposure to a novel noisy stimulus, in the form of an electric hair clipper, during the Handling condition but not in the Neutral condition). Moreover, some traits could not be fully expressed in certain conditions. For example, ‘Protective’, ‘Friendly/Social’ and ‘Solitary’ may have been difficult to assess during the Handling condition because individuals were physically isolated and unable to interact with conspecifics, whereas these traits were observable in the Neutral and Feeding conditions. Their exclusion was further supported by the factorial analyses: ‘Protective’, ‘Friendly/Social’ and ‘Solitary’ showed low MSA values, whereas ‘Fearful’ generated a potential Heywood case. These findings suggest that, although these traits may be relevant to addax personality, they were not suitable for the factorial structure under the conditions of the present study and should be considered in future assessments designed to allow their expression across contexts. Together with previous ethograms describing locomotor, feeding, aggressive and sexual behaviour [57,58,79], our findings expand the behavioural characterization of addax antelopes and provide a framework for personality assessment in this species. Addax antelope is critically endangered [43] and is currently mostly maintained in captivity, where handling and husbandry procedures are frequent. Handling-related procedures in non-domestic ungulates have been associated with behavioural indicators of distress (e.g. increased vigilance, vocalizations, agonistic interactions and escape behaviour) as well as physiological stress responses, including elevations in glucocorticoids, heart rate, respiratory rate and body temperature [30,80,81]. In this context, a better understanding of interindividual variation in handling responses could support refined husbandry and management practices and reduce adverse outcomes [82]. Moreover, addax antelope reintroductions have been conducted previously [47,83] and may be increasingly required given the ongoing decline of wild populations. Personality has been proposed as a relevant factor influencing reintroduction outcomes [13,14], including survival [84] and reproductive success [85]. Integrating personality assessments into captive management and future translocation or reintroduction programmes appears highly advisable for this species. Accordingly, by identifying consistent individual differences in responses to handling, our findings provide information that may improve captive management practices and contribute to the assessment of behavioural competence, a key consideration alongside genetics, welfare and demography in IUCN reintroduction guidelines [86].

Based on behavioural items, we identified four personality axes, which are in line with some of the commonly reported axes in ungulates [3,87] and other vertebrates [2,15,16]. ‘Aggressive/Excitable’ axis was characterized by high aggressiveness and excitability, together with persistence and vigilance, and low calmness and submission; thus, it broadly aligns with the ‘Aggressiveness’ axis as described in the literature [2]. The ‘Active/Unpredictable’ axis was primarily defined by higher scores of ‘Active’, ‘Inquisitive’ and ‘Erratic’ items and lower scores of ‘Slow’, ‘Inactive’ and ‘Predictable’ items. This axis partly overlaps with activity-related dimensions and shares features with boldness, as bold individuals are often described as curious and are prone to approach and remain near novel objects while showing low tension [4,54]. The ‘Confident’ axis was mainly driven by the item ‘Confident’, with a moderate negative association with ‘Submissive’ and ‘Socially Anxious’ items and generally low activity, excitability and aggressiveness. Therefore, Confidence was not coupled with Aggressiveness in addax antelopes, unlike reports in red deer (*Cervus elaphus*) [54]. Finally, the ‘Bully’ axis reflected a tendency to dominate conspecifics through non-physical interactions, remain close to others (‘Popular’) and be more focused (low ‘Distractible’) alongside social anxiety. Given that Sociability can be defined as the motivation to remain close to conspecifics [88], individuals with higher scores on ‘Bully’ axis may show greater Sociability than ‘Confident’ individuals. In summary, ‘Aggressive/Excitable’ and ‘Active/Unpredictable’ showed clearer parallels with established personality dimensions, whereas ‘Bully’ and ‘Confident’ may represent more species-specific or context-dependent constructs in addax.

Most personality axes were associated with interindividual variation in behavioural and physiological responses to handling, both during the procedure (body surface temperature and exit velocity) and over subsequent days (grazing behaviour). This occurred despite the animals being accustomed to human presence, familiar with the facilities and the minimally invasive handling procedures performed, which highlights the importance of continuously improving understanding of the effects of handling under captive non-domestic species. Also, these findings are consistent with previous studies linking personality to consistent interindividual differences in foraging and locomotor behaviour, as higher boldness has been associated with greater food intake [4] and shorter flight response distances to the presence of humans [87]. Similarly, Yang *et al.* [89] found that bold musk deer (*Moschus berezovskii*) individuals showed greater exploratory behaviour, including more frequent approaching and sniffing of novel stimuli, shorter distances to the stimulus and a higher tendency to maintain routine behaviours such as feeding, resting and rumination under potentially stressful conditions, than shy individuals. Higher scores in ‘Active/Unpredictable’ axis were associated with lower exit velocity and a greater increase in grazing within 48 h post-handling. SAM axis activation results in catecholamine release and short-term increases in locomotion, as part of the fight-or-flight response, which is typically accompanied by parasympathetic suppression [18–20]. Thus, a slower exit from the crush suggests a moderate flight response in animals with higher ‘Active/Unpredictable’ ratings. Conversely, maintained or increased parasympathetic activity has been linked to higher feed intake [90]. Subsequent activation of HPA axis leads to cortisol secretion, which can stimulate feeding at moderate levels but suppress appetite at high concentrations [91–93]. Although FCM did not differ across individuals with different ‘Active/Unpredictable’ scores, the observed behavioural pattern seems consistent with relatively lower sympathetic activation and a greater involvement of parasympathetic and HPA-mediated responses. In summary, the locomotor and feeding responses associated with the ‘Active/Unpredictable’ axis suggest a stress profile characterized by reduced SAM activation and a comparatively stronger parasympathetic/HPA contribution.

Higher ‘Bully’ scores were associated with lower exit velocity and consistently higher surface temperatures (eye, shoulder and rump). Lower exit velocity suggests reduced SAM activation, which aligns with the physiological effects of sympathetic arousal, including peripheral vasoconstriction and decreased skin and eye temperatures [64,94]. Thus, the combination of low exit velocity and higher surface temperature in individuals with higher ‘Bully’ scores may reflect a weaker sympathetic response to handling. In contrast, the higher exit velocity and lower shoulder temperature observed in individuals with higher ‘Aggressive/Excitable’ scores are consistent with a stronger sympathetic stress response.

A cautious interpretation is required regarding the specific branch of the autonomic nervous system that underlies, in particular, changes in surface temperature, as the literature reports inconsistent patterns. For example, both increasing [95,96] and decreasing [64,94] eye temperature responses were described under stress conditions. Furthermore, peripheral temperature response may reflect the interaction of multiple neuroendocrine mediators (catecholamines, dopamine and dopa) and receptor-specific effects [97–99]. In this sense, the present findings provide a valuable starting point for future studies aimed at elucidating the autonomic nervous system's contribution to stress responses in the addax under captive conditions.

Personality axes were associated with distinct behavioural profiles and handling responses. Under stressful conditions, individual variation can be described along a continuum from proactive to reactive coping styles [17]. Proactive individuals typically exhibit more active coping strategies, greater aggression and routine formation, and a defence response biased towards a fight-or-flight strategy [20]. In contrast, reactive individuals generally display greater behavioural flexibility, lower aggression and more passive or cautious responses to challenges. In our study, individuals with higher ‘Aggressive/Excitable’ scores were consistently excitable, aggressive and persistent, also during handling, when they showed higher exit velocity and decreased body surface temperature. These responses may be consistent with greater acute sympathetic activation during handling; together with their aggressive and persistent behavioural profile, which suggests that this axis may be associated with a more proactive coping style [17,22,23]. Conversely, individuals with higher ‘Active/Unpredictable’ and ‘Bully’ scores displayed non-aggressive patterns, and individuals with higher ‘Active/Unpredictable’ scores were particularly inquisitive and erratic, consistent with higher neophilia. These characteristics may reflect a more flexible response to challenging situations and therefore show some similarities with traits commonly associated with a reactive coping style [17].

Responses to handling were consistent and complementary across physiological and behavioural measures, allowing us to propose coping-style patterns associated with the identified personality axes. Future studies incorporating additional physiological (e.g. thermal response, heart rate, haematological and biochemical traits, and catecholamine production) and behavioural variables (e.g. vocalizations, self-directed behaviour, urination and defecation) would help disentangle coping style from stress reactivity and improve our understanding of how both dimensions relate to personality traits in addax antelopes. Besides, the absence of personality-related differences in FCM may partly reflect uncertainty regarding the temporal relationship between cortisol secretion and the appearance of its metabolites in faeces, as samples were collected within 48 h after handling. Gaining a deeper understanding of how cortisol production is reflected in variations of its metabolites in faeces appears essential in this regard.

## 5. Conclusion

We concluded that captive *A. nasomaculatus* antelope exhibited four personality traits linked to distinct handling responses, with ‘Active/Unpredictable’ and ‘Bully’ individuals showing lower exit velocity and higher surface temperature, whereas ‘Aggressive/Excitable’ individuals showed the opposite pattern. It contributes to the still limited literature on personality in non-domestic ruminants, which remains largely concentrated on cervids, with comparatively fewer studies in other taxa. By revealing consistent links between personality and handling responses, our results support the inclusion of individual behavioural differences in decision-making processes aimed at improving the management and long-term conservation of this species.

## Data Availability

The data and code can be accessed by an Open Science Framework repository [100].
